# Mechanism of Continuous Melting and Secondary Contact Melting in Resistance Heating Metal Wire Additive Manufacturing

**DOI:** 10.3390/ma13051069

**Published:** 2020-02-28

**Authors:** Chengwei Yuan, Shujun Chen, Fan Jiang, Bin Xu, Shanwen Dong

**Affiliations:** 1 Engineering Research Center of Advanced Manufacturing Technology for Automotive Components, Ministry of Education, College of Mechanical Engineering and Applied Electronics Technology, Beijing University of Technology, Beijing 100124, China; bj_ycw@126.com (C.Y.); sj_chen@bjut.edu.cn (S.C.); cougarbin@163.com (B.X.); bjycw@126.com (S.D.); 2 Beijing Engineering Researching Center of laser Technology, Beijing 100124, China

**Keywords:** resistance heating, continuous melting, dynamic resistance, temperature distribution

## Abstract

Resistance heating metal wire materials additive manufacturing technology is of great significance for space environment maintenance and manufacturing. However, the continuous deposition process has a problem in which the metal melt is disconnected from the base metal. In order to study the difference between the second contact melting of the disconnected metal melt and the continuous melting of the metal wire as well as eliminate the problem of the uneven heat dissipation of the base metal deposition on the melting process of the metal wire, the physical test of melting the metal wire clamped by the equal diameter conductive nozzle was carried out from the aspects of temperature distribution, temperature change, melting time, dynamic resistance change, and the microstructure. The current, wire length, and diameter of the metal wire are used as variables. It was found that the dynamic resistance change of the wire can be matched with the melting state. During the solid-state temperature rise, due to the presence of the contact interface, the continuous melting and secondary contact melting of metal wires differ in dynamic resistance and the melting process. The continuous melting of the metal wire was caused by the overall resistance of the wire to generate heat and melt, and the temperature distribution is “bow-shaped”. In the second contact melting, the heat generated by the contact interface resistance was transferred to both ends of the metal wire to melt, and the temperature distribution is “inverted V”. The microstructure of the metal wire continuous melting and secondary contact melting solidification is similar. The continuous melting length of the metal wire is greater than the melting length of the secondary contact.

## 1. Introduction

In-space metal materials’ additive manufacturing technology has been applied to the manufacture and repair of metal parts in the orbit. It will become an important research direction in the future aerospace manufacturing field [1,2,3]. At present, the heat sources used in metal additive manufacturing technology are mainly laser beams, electron beams, plasma beams, and arc beams [4,5]. Apart from the ground environment, the space environment poses severe challenges for metal additive manufacturing technology, including the current heat transfer system, the additive manufacturing process, and heat source equipment [6,7]. Metal wire additive manufacturing technology using a laser beam as a heat source has the advantages of high energy density, high precision, high efficiency, and high flexibility [8,9]. However, in this approach, the device structure is relatively complicated, the power output is high, and the energy utilization rate is relatively low. Compared with the laser heat source, the electron beam heat source has higher energy utilization. However, the device structure is also relatively complicated in this approach [10,11]. Wire additive manufacturing technology using the plasma beam as a heat source have the characteristics of high heat input, high utilization rate, high manufacturing efficiency, and rapid manufacturing [12,13]. However, the arc is difficult to ignite due to the influence of the low-pressure environment in space. Our research group proposed a metal manufacturing method suitable for space environments and resistive heating metal wire additive manufacturing technology [14]. The electrical energy is converted into Joule heating by the current, and the wire was melted and shaped. This method has the advantages of high thermal efficiency, precise heat input control, and simple additive manufacturing equipment [15,16,17].

Yu Zhang et al. [18] designed two different resistance spot welding joint structures, established a thermo-electric coupling finite element model, and analyzed the temperature fields of the two joints. The temperature field and microstructure characteristics of these two joints were studied through simulation and experiments. Kang Zhou et al. [19] found that the electrical resistivity of the molten nugget was theoretically and experimentally deduced, according to detailed theoretical analyses based on the principle of electrical resistivity and the resistance spot welding process. Yi Luo et al. [20] calculated the dynamic resistance based on the welding current and electrode voltage detected in resistance spot welding and studied the thermal effects of nugget growth during nugget forming. The growth process of nuggets in resistance spot welding can be divided into three stages. Wang Xiaoyu et al. [21] systematically studied the effects of melting time and temperature on the microstructure and the resulting electrical and heat transfer characteristics. With the increase of melting time, the microstructure of the samples will be significantly improved. The above researchers analyzed the spot-welding process through temperature field, dynamic resistance, microstructure, and more. Compared with spot welding, the heat source of resistance heating metal wire additive manufacturing is the same. However, the filler of resistance heating metal additive manufacturing is the metal wire. There is no external force loading. It needs continuous heating and transition when it is applied to additive manufacturing.

The continuous deposition process has the problem that the metal melt is disconnected from the base metal. In order to study the difference between the second contact melting of the disconnected metal melt and the continuous melting of the metal wire. The influence of uneven heat dissipation of the base metal deposition on the melting process of the metal wire is avoided. The physical test of the melting of the metal wire clamped by the equal diameter conductive nozzle is designed. The current, wire length, and diameter of the metal wire are used as variables. The research is carried out from the aspects of temperature distribution, temperature change, melting time, dynamic resistance change, and microstructure.

## 2. Resistance Heating Wire Additive Manufacturing Principle

**Figure 1:**
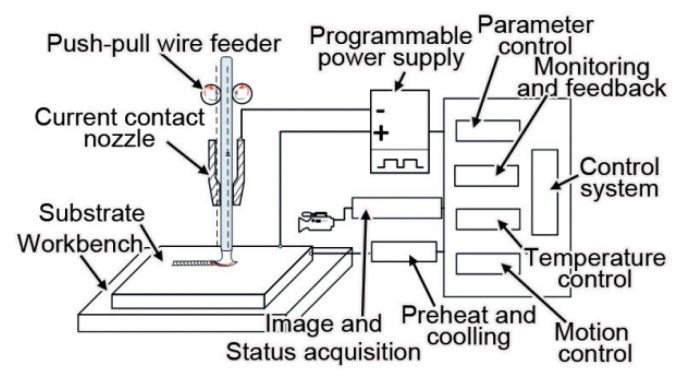
The experimental setup for resistance heating metal wire additive manufacturing.

**Figure 2:**
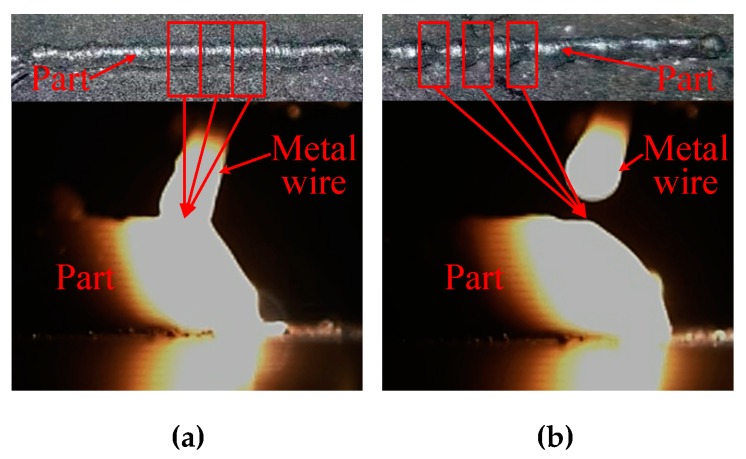
The morphology of resistance heating metal wire additive manufacturing. (**a**) Continuous melting and (**b**) disconnected melt.

Our research group proposed a metal manufacturing method suitable for the space environment and resistance heating metal wire additive manufacturing technology. Figure 1 shows the experimental setup for resistance heating metal wire additive manufacturing. The wire feeder is short circuited between the metal wire and the base plate and the current generates Joule heat through the short-circuited wire and substrate. The preliminary study has been performed by our research group on the additive manufacturing technology of resistance heating wire by dragging the metal wire to achieve additive manufacturing of metal parts. Experiments have proved the feasibility of resistance heating wire manufacturing in the space environment [14]. It was found that, in the continuous deposition manufacturing process, once the metal wire and the substrate are disconnected, the melt contacts the matrix again and the heat production will change, which will affect the melting process of the wire. The research found that, in the continuous deposition manufacturing process, there is a problem of disconnecting the metal wire from the substrate. Moreover, the contact of the molten pool with the substrate again will cause a change in heat generation, which will affect the melting process of the wire, as shown in Figure 2.

**Figure 3:**
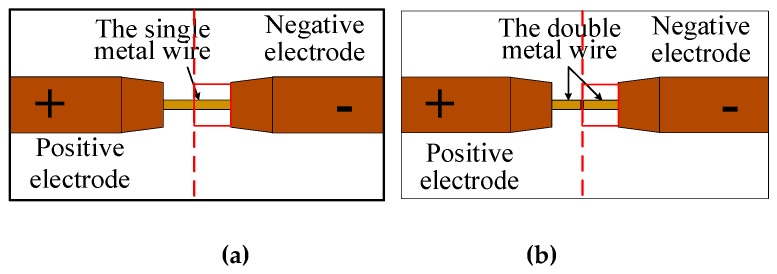
The simplified physical model of the resistance heating metal wire additive manufacturing. (**a**) A single metal wire and (**b**) double metal wires.

In the continuous manufacturing process of resistance heating wire, the different shapes of the base metal deposition affect the heat dissipation during the manufacturing process. Different shapes of the base metal deposition have different heat dissipation speed, which leads to uneven heat dissipation. The continuous deposition process has a problem in which the metal melt is disconnected from the base metal. In order to study the difference between the second contact melting of the disconnected metal melt and the continuous melting of the metal wire, the influence of uneven heat dissipation of the base metal deposition on the melting process of the metal wire is avoided. The physical test of the melting of the metal wire clamped by the equal diameter conductive nozzle was designed. Figure 3 is a simple physical model of the forming process of resistance-heated wire. The base metal and the current contact nozzle are simplified as positive and negative electrodes, respectively. When the metal wire is continuously melted, the metal wire and the base metal deposition are fused together and regarded as the same metal body. The metal wire and the deposition part are simplified as a single metal wire. There is a contact interface between the metal wire and the deposition part during the second contact of the disconnected metal melt. The metal wire and the deposition part are simplified as the double metal wires. In the simplified experimental model, the heat generation of the metal wire and the contact interface are unchanged. The shape of the base metal deposition does not affect the purpose of the experimental study.

## 3. Materials and Experimental Procedure

### 3.1. Experimental System

**Figure 4:**
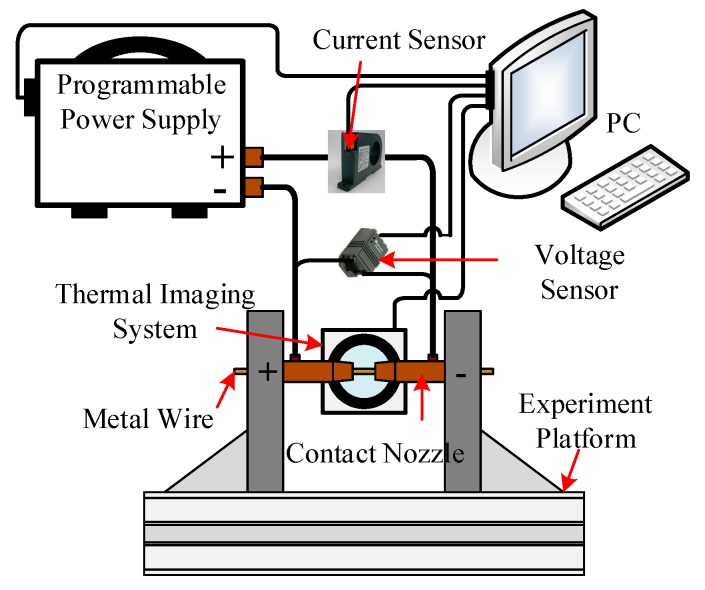
Resistance heating single and double metal wire experimental system.

Figure 4 shows the experimental system for the resistance heating single and double metal wire melting process. The metal wire passes through two coaxial current contact nozzles. The programmable power source outputs current. The short-circuit current flows through the metal wire body to generate Joule heat, which accumulates heat and the metal wire melts. Optris PI Xi 400 (Optris GmbH, Berlin, Germany) was used to collect the temperature changes during the melting process of the wire. Voltage sensor CHV-25P (Beijing SENSOR Electronics Co., Ltd., Beijing, China) and current sensor CHB-500S (Beijing SENSOR Electronics Co., Ltd., Beijing, China) were used to collect the change of the electrical signal during the melting process of metal wire. The computer synchronously collects the electrical signal and temperature change of the metal wire melting process through the National Instruments acquisition card. The frame frequency of the thermal imager is 1000 fps, the temperature range is 600 °C ~ 1800 °C, the voltage measurement range of CHV-25P (Beijing SENSOR Electronics Co., Ltd., Beijing, China) module is ± 25V, and the measurement range of CHB-500S (Beijing SENSOR Electronics Co., Ltd., Beijing, China) current sensor is ± 500A.

### 3.2. Experimental Principle

The short-circuit current flows through the wire to generate Joule heat, which accumulates and the temperature of the wire increases. The temperature change mainly depends on the short-circuit current, electrode distance, the diameter of the wire, and its physical characteristics [22]. According to the principle of resistance heating metal, the resistance (R) of metal wire in the heating process is shown below.

(1) $$R=\frac{\rho L}{\pi r^{2}},$$

where ρ is the resistivity of Q235, *L* is the length of the heated wire, and *r* is the radius of the heated wire. The resistance is proportional to the length of the wire and inversely proportional to the square of the radius of the wire. Joule heat (W) is generated by the current through the metal wire material, which is obtained by Joule’s law [23].

(2) $$W=\int_{0}^{t_{m}}i{\left(t\right)}^{2}R\left(t\right)dt,$$

where *i*(*t*) is the instantaneous current of the wire, *r*(*t*) is the resistance of the wire, and *t_m_* is the melting time of the wire. The relationship between current and resistance heat is quadratic, and the relationship between resistance and heat is linear.

The other part of the heat is generated by the contact resistance between the conductive nozzle and the contact surface of the metal wire material. When the current flows through the metal wire, joule heat is generated on the contact surface, and part of the heat is transmitted to the metal wire material. The Joule heat generated on the contact surface of the conductive nozzle and the metal wire material is directly proportional to the heat conductivity of the contact surface. The resistance heat generated by the contact resistance between the conductive nozzle and the metal wire is shown below [24].

(3) $$Q_{c}=\int_{0}^{t_{m}}\frac{K_{1}}{K_{1}+K_{2}}i{\left(t\right)}^{2}R_{c}\left(t\right)dt,$$

where *R_c_*(*t*) is the contact resistance between the contact nozzle and the metal wire, which is small and can be regarded as the constant. K_1_ and K_2_ are the thermal conductivity between the nozzle and the metal wire. The heat loss of wire from heat conduction to the conductive nozzle (*Q_h_*) is shown below [25].

(4) $$Q_{h}=h_{c}A_{c}\left(T_{2}-T_{1}\right),$$

where *h_c_* is the thermal conductivity of the contact surface between the metal wire and the conductive nozzle, *A_c_* is the contact area of the metal wire and the conductive nozzle, and *T_2_* and *T_1_* are the temperatures of the metal wire and the temperature of the conductive nozzle, respectively. Argon gas protects the metal wire from being heated. The heat (Q1) lost by the outer surface of the metal wire through heat radiation is shown below [26].

(5) $$Q_{1}=\varepsilon \sigma_{0}A_{r}\left(T_{2}^{4}-T_{3}^{4}\right),$$

where *ε* is the surface emissivity, *σ*_0_ is the Stefan constant, *A_r_* is the radiation area of the wire, and *T_3_* is the temperature of the shielding gas. According to the law of energy conservation, the heat of the heat (Q) of the metal wire during heating can be expressed by the equation below.

(6) $$Q=W+Q_{c}-Q_{h}-Q_{1}.$$

### 3.3. Materials and Experimental Method

This study uses the most common metal material Q235 steel. The metal wire is made by advanced electroplated copper technology with a strong deoxidation, which can reduce the oxygen content in the melting process and ensure the quality of the Joule heating metal wire melting. All samples were cleaned with acetone by the ultrasonic cleaner before the experiment. The metal wire was installed between two coaxial electrodes and heat-treated in 500 °C air for one hour to release the residual stress produced by processing and drawing.

The continuous deposition process has the problem that the metal melt is disconnected from the base metal. In this article, in order to study the difference between the second contact melting of the disconnected metal melt and the continuous melting of the metal wire. The current, wire length, and diameter of the metal wire are used as variables. The research was carried out from the aspects of temperature distribution, temperature change, melting time, dynamic resistance change, and microstructure. The differences between the continuous melting process of the metal wire and the secondary contact melting process of the metal melt were analyzed. The specific scheme was described below.

A constant current of 60 A, 80 A, and 100 A was chosen respectively to study the effect of the different current on the experiment. The specific experimental parameters are shown in Table 1 (1–1, 1–2, and 1–3). Moreover, the electrode distance of 4 mm, 6 mm, 8 and mm was chosen, respectively, to study the effect of the electrode distance on the experiment (Table 1 (2–1, 2–2, and 2–3)). In order to study the effect of the wire diameter on the experiment. The diameters of the metal wires are 0.8 mm, 1.2 mm, and 1.6 mm, respectively (Table 1 (3–1, 3–2, and 3–3)). The current flows through the double metal wires in coaxial contact, which is helpful to study the effect of the secondary contact interface after the disconnection of the metal melt in the experiment. The lengths of the metal double wire are 2 mm and 2 mm, 3 mm and 3 mm, and 4 mm and 4 mm, respectively. The specific experimental parameters (Table 1 (4–1, 4–2, and 4–3)).

## 4. Results and Discussion

### 4.1. Temperature Field Distribution of Metal Wire

The current passed through the wire and released Joule heat. Zhao et al. analyzed the steady-state temperature distribution of the current through the conductor. Assuming that the properties of the material have nothing to do with temperature, we can get the temperature distribution of the metal wire [27].

(7) $$T=\int_{0}^{l}\left[\left(T_{0}-T_{\infty }-\frac{\rho_{e}AJ^{2}}{ch_{c}}\right)\frac{cosh\omega x}{\cosh\left(\frac{\omega L}{2}\right)}+T_{\infty }+\frac{\rho_{e}AJ^{2}}{ch_{c}}\right]dl,$$

where *T*_0_ is the temperature at the end of the wire. t_∞_ is the ambient temperature (i.e., the temperature far from the surface of the metal wire). *A* is the cross-sectional area of the wire. J is the current density. *c* is the sum of the surface area per unit length. *ρ_e_* is the resistivity. *h_c_* is the heat transfer coefficient, and K in $\omega^{2}=ch_{c}/kA$ is the heat conductivity coefficient.

**Figure 5:**
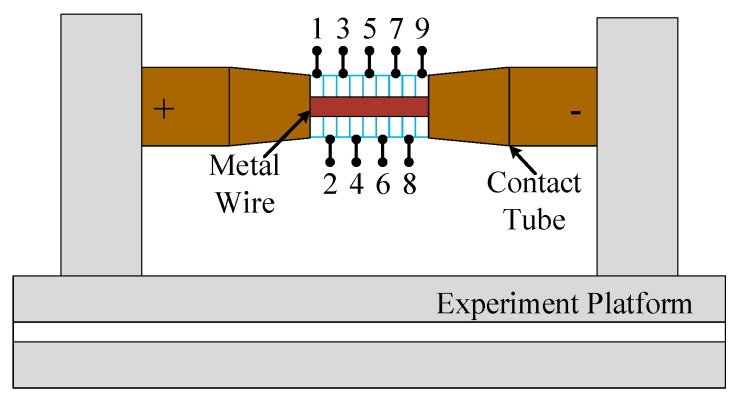
Schematic diagram of metal wire temperature distribution at different positions.

Figure 5 is the schematic diagram of the temperature distribution of the metal wire at different positions. The image of the melting process of the metal wire collected by the thermal imager was divided into nine sections. The temperature of each section of the metal wire at different times was extracted to obtain the temperature distribution of the metal wire at different positions. It is seen from the calculation of Equation (7) that the error between the experimental result and the theoretical calculation is small.

**Figure 6:**
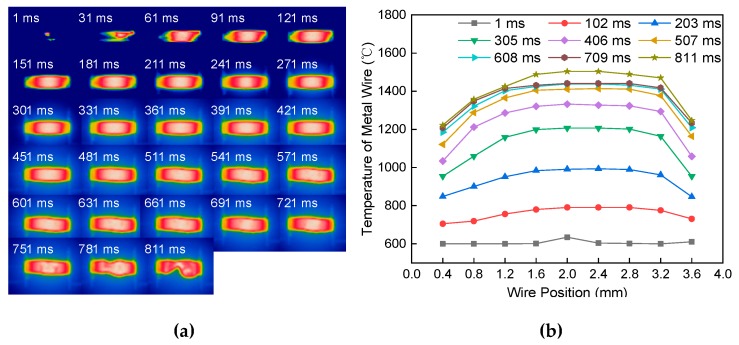
Effect of current on melting of a single metal wire. (**a**) Melting process. (**b**) Temperature distribution.

Figure 6a shows the melting process image of the single metal wire at a constant current of 60 A passing through the length of 4 mm and a diameter of 1.2 mm. It is seen from the image that, in the early stage of heating, the temperature is higher in the middle of the metal wire. While the heat is accumulated in the middle stage of heating, the temperature in the middle of the metal wire has gradually increased. The high-temperature area gradually increased. The heat of the metal wire was conducted in a strip along the axial direction of both ends of the electrode, which led to the temperature of the metal wire gradually increasing. At the end of heating, the high-temperature region in the middle of the wire increases. The melted wire deforms flexibly, which forms necking at the highest temperature region. Figure 6b shows the temperature distribution of the metal wire at different positions and different times. It is seen from the figure that, before 100 ms, the temperature distribution is relatively uniform in the middle section and both ends of the metal wire. With the increase of time, the temperature distribution of the metal wire is in a “bow-shape”. The electric extreme temperature is low while the temperature in the middle part is high. The temperature difference between the two ends and the middle of the metal increased gradually.

**Figure 7:**
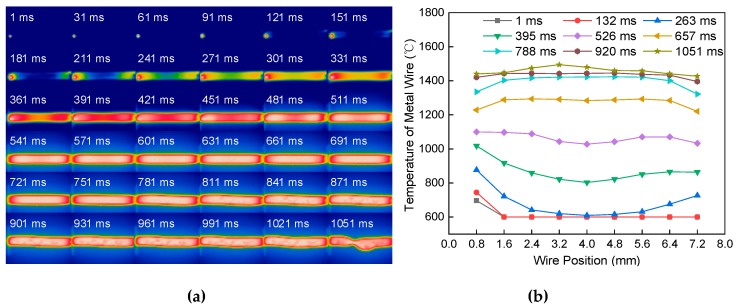
Effect of electrode distance on melting of a single metal wire. (**a**) Melting process. (**b**) Temperature distribution.

Figure 7a shows the melting process of the single metal wire at a constant current of 60 A passing through a length of 8 mm and a diameter of 1.2 mm. It is different from the heat transfer mode in Figure 6a. At the initial stage of heating, the temperature of the metal wire near the end of the electrode begins to rise. In the middle of heating, the heat accumulated and transmitted from both ends of the electrode to the middle of the wire. The temperature of the wire tends to balance gradually. At the end of the heating, the temperature of the wire reaches the melting point temperature. The necking was formed in the highest temperature region. Figure 7b shows the temperature distribution of the metal wire at different positions and different times. During 0–540 ms, the temperature distribution is low in the middle of the wire and high in both ends of the wire. During 540–810 ms, the temperature distribution is high in the middle of the wire and low at both ends of the wire. After 940 ms, the temperature distribution of the metal wire is balanced.

**Figure 8:**
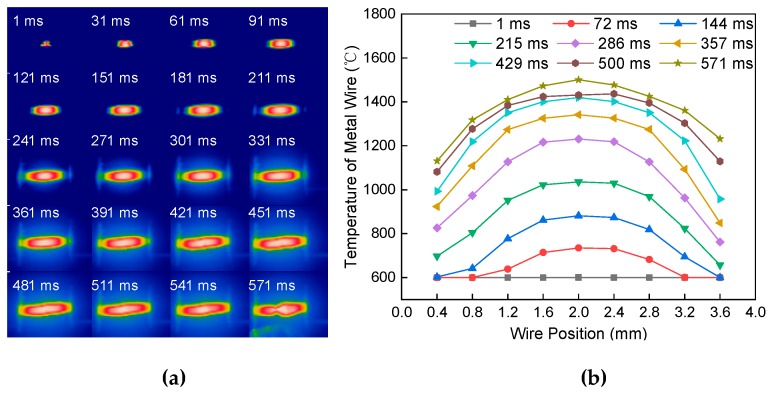
Effect of wire diameter on the melting of single metal wire. (**a**) Melting process. (**b**) Temperature distribution.

Figure 8a shows the melting process of the single wire at a constant current of 60 A passing through the length of 4 mm and a diameter of 0.8 mm. The heat transfer mode is similar to Figure 6a. However, the wire with a diameter of 0.8 mm has a faster heating speed. The temperature difference between the wire near both ends of the electrode and the middle part of the wire is large. Figure 8b shows the temperature distribution of the metal wire at different positions and different times. The temperature distribution is “bow-shaped”. With the increase of time, the temperature difference between the middle and both ends of the metal wire increased gradually.

**Figure 9:**
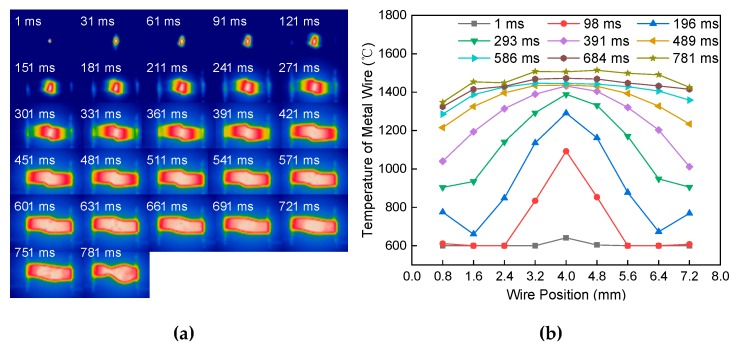
Effect of wire length on the melting of double metal wires. (**a**) Melting process and (**b**) temperature distribution.

Figure 9a shows the 60 A constant current passing through the double metal wires with a length of 4 mm and a diameter of 1.2 mm. At the initial stage of heating, there are burrs and sharp edges between the two wires, which result in less contact area than the cross-sectional area of the metal wire. The instantaneous heat production is higher than in the stable stage. In the middle stage of heating, the heat of the metal wire is accumulated. The heat of the metal wire is transmitted from the contact position of the double wire along the axial direction of the metal wire to the electrode direction. The temperature of the metal wire increases. At the end of heating, the wire began to melt and necking occurred at the highest temperature. Figure 9b shows the temperature distribution of the metal wire at different positions and times. At a time of 0–97 ms, the temperature distribution of the wire is an “inverted-V-shape" in the middle of both ends. The temperature at both ends of the wire is less than or equal to 600 °C. At the time of 97–288 ms, the temperature distribution of the wire was “W-shaped”. With the increase of time, the temperature difference between the middle and both ends of “W-shaped" increased gradually. After 288 ms, the temperature distribution of the wire is “bow-shaped”.

The main factors affecting the temperature distribution of the metal wire are the short-circuit current, the length, and the diameter of the metal wire. The temperature distribution of continuous melting and secondary contact melting is different. The temperature distribution during the melting of the continuous melting process is “bow-shaped”. The temperature distribution during the melting of the secondary contact melting process first showed an “inverted V-shape”, then the “W-shape”, and, lastly, the “bow-shaped" change.

### 4.2. Factors Affecting Wire Temperature

**Figure 10:**
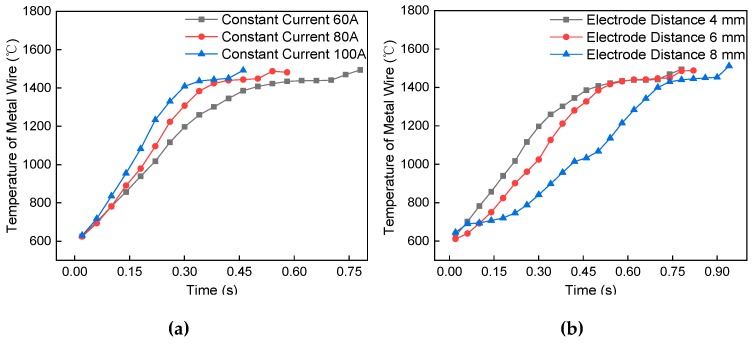

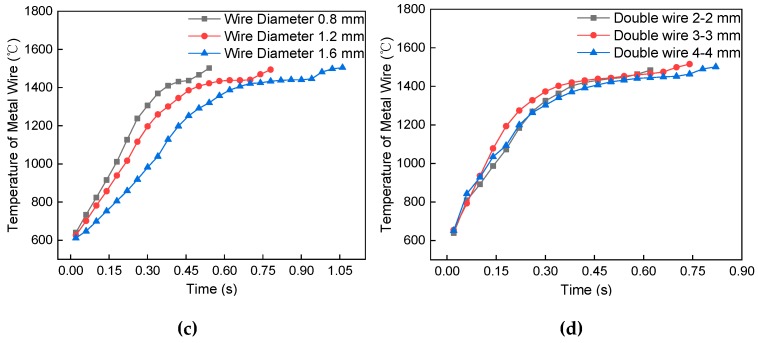
Resistance heating wire temperature trend. (**a**) Different short-circuit current, (**b**) electrode distance, (**c**) metal wire diameter, and (**d**) double metal wire length.

During the electro-thermal test, the short-circuit current passes through the wire and measures the change of wire temperature with time. Figure 10 shows the temperature change curve of the resistance heating wire melting process. With the increase of time, the temperature of the wire increases linearly, and, after the transient, the local temperature reaches a constant value. Figure 10a shows the effect of different current values on the temperature change of the wire. With the increase of a short-circuit current, the slope of temperature rise gradually increases. The time to achieve local heat balance gradually decreased, which is due to a large amount of Joule heat being released when the current with high current density passes through the wire. Figure 10b shows the effect of temperature change of the wire electrode distance. With the increase of wire length, the slope of temperature rise gradually decreases. The time to achieve local heat balance gradually decreases. This is because the increase of wire length leads to the increase of heating objects and the decrease in heating speed. After reaching the heat balance, the local heat holding time will decrease. Figure 10c shows the trend of temperature rise of metal wire with different diameters. As the diameter of the metal wire decreased, the temperature increase rate of the wire gradually increased, and the time to achieve local thermal equilibrium decreased. This is due to the decrease in the diameter of the wire leads to an increase in resistance, and the resistance value determines the heat generated by the metal wire. Figure 10d shows the temperature rising trend of the double metal wires of different lengths. The temperature of the metal wire first increased rapidly and then increased slowly. The speed of the temperature increase of the metal wires gradually decreased with an increasing length of the double metal wires, and the time to reach the local thermal equilibrium gradually increased. This is because the burrs and sharp changes at the interface of the double metal wires resulting in faster temperature rise for the wire. With the increase of the metal wire length, the increase of heat conduction distance leads to the increase of the heat balance time.

### 4.3. Evolution of Electric Resistance

**Figure 11:**
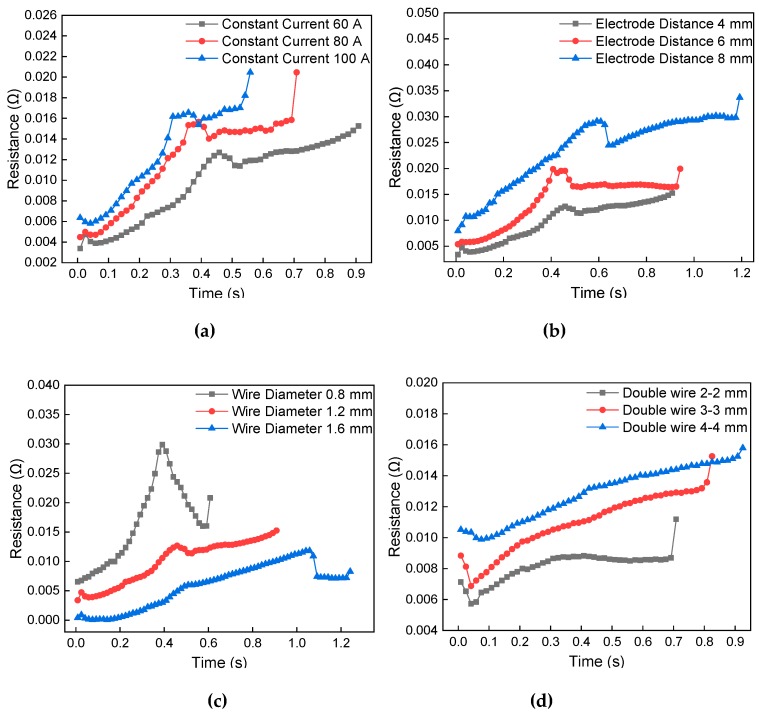
Dynamic resistance change of metal wire during melting. (**a**) Different short-circuit current; (**b**) electrode distance, (**c**) metal wire diameter, and (**d**) double metal wire length.

By measuring the potential difference at both ends of the electrode, the resistance of the wire at both ends of the electrode can be calculated. Figure 11 shows the trend of the dynamic resistance during the melting process of the wire. There is a corresponding relationship between the resistance value and the temperature of the metal wire. The increase of local temperature results in the change of wire resistivity. The temperature of the metal wire increases linearly, and the resistance increases with the rise of the temperature. The temperature of the metal wire reaches the solid temperature, and the resistance rises to the maximum value. The temperature of the wire continues to rise, the wire begins to melt, and the resistance value begins to drop to a certain value and remains stable. When the temperature of the wire reaches the melting point, the necking of the melt occurs, and the resistance gradually increases to infinity. The linear increase speed of resistance value is due to the increase in temperature and surface oxidation. The stable increase of resistance value is due to the transformation of the partial metals from solid to liquid. Figure 11a–c shows that the changing trend of resistance in the melting process of metal wire under the action of the different current, different length, and different diameters. The changing trend of resistance is approximately the same. Both increasing the short-circuit current and decreasing the wire diameter can be considered as changing the current density. With the increase of current density, the resistance of the metal wire increases gradually. With the increase of wire length, the total resistance will increase. Figure 11d shows dynamic resistance during the melting process of the double metal wire. There are burrs and sharp edges between the wires at the initial stage of heating, and the contact resistance is large. As the temperature of the wire increased, the resistance gradually decreased to a certain value. The temperature of the wire begins to rise and the resistance increases linearly until the current fails. With the increase of the double metal wire length, the slope of the resistance increases gradually. The short-circuit current provides energy for the melting of metal wire. The temperature of the metal wire has a more significant corresponding relationship with the dynamic resistance.

The dynamic resistance heat is an important evaluation index for measuring the thermal effect of metal wires. It is closely related to the molten state of the metal wire. In the initial stage of heating, the difference between continuous melting and secondary contact melting dynamic resistance changes is the largest. This is because the contact resistance of the secondary contact interface needs to be eliminated. According to the dynamic resistance change trend, the melting state of the metal wire can be monitored to avoid the occurrence of melt disconnection.

### 4.4. Melt Fracture Time

From the change of resistance (voltage) to time, we can determine the current failure time of the metal wire (the metal wire is fused) when it bears the current with high current density. From Equations (1) to (6), the melting time of the metal wire can be obtained.

(8) $$t=\frac{Cm∆T+h_{c}A_{c}\left(T_{2}-T_{1}\right)+\varepsilon \sigma_{0}\left(T_{2}^{4}-T_{3}^{4}\right)}{i^{2}R+\frac{K_{1}}{K_{1}+K_{2}}i^{2}R_{c}},$$

**Figure 12:**
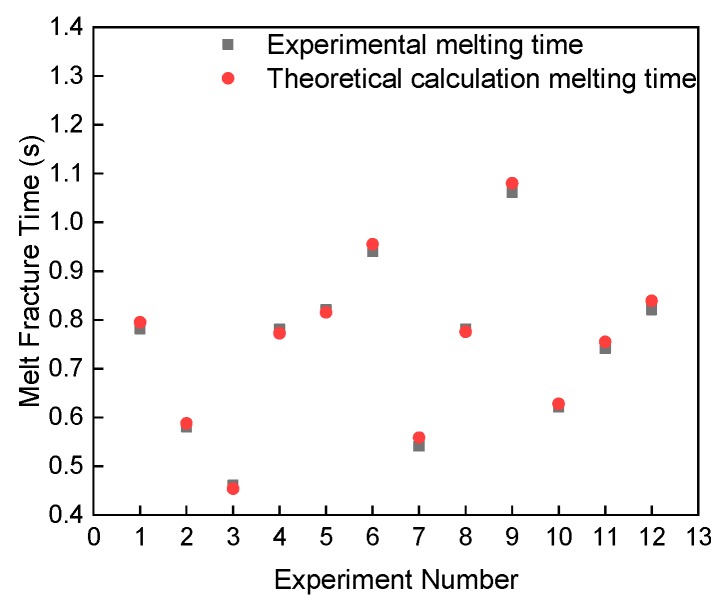
Relationship between metal wire melting experiment and theoretical calculation.

Figure 12 shows the relationship between the melting experiment and theoretical calculation. The variables with serial numbers of 1–3, 4–6, 7–9, and 10–12 in the figure are the melting time of different short-circuit current, different electrode distance, different wire diameter, and different double wire length. The error between the experimental and theoretical calculation time is about 2%, 2%, 4%, and 7%, respectively. The error between the melting time of the single wire and the theoretical calculation time is small, while the error of the double wire is large. The theoretical calculation ignores the burr and the sharp edge between the double wires. The time for continuous melting and secondary contact melting can be calculated by theory. Moreover, the additive manufacturing time of metal parts can be estimated by a theoretical calculation.

**Figure 13:**
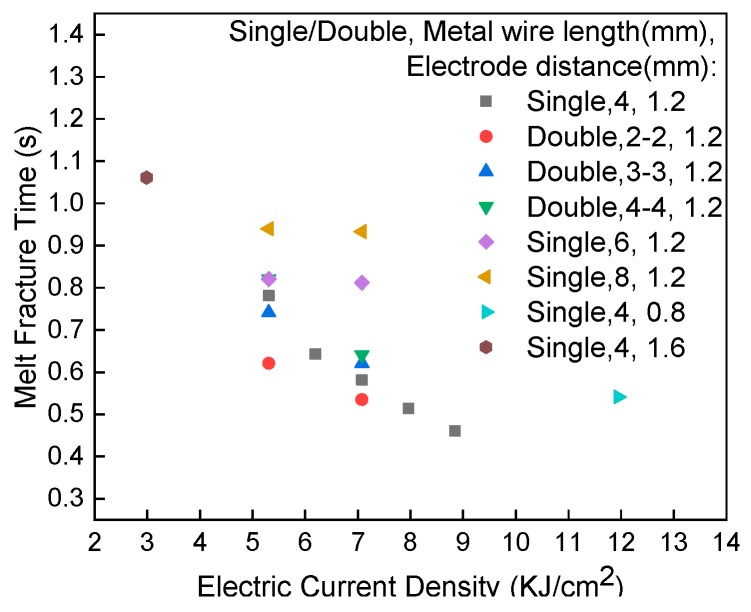
Dependence of the time-to-fusing on electric current density.

Figure 13 shows the dependence of the fusing time of the metal wire on the current density. The fracture time increased with the rise of the current density because the local temperature increases with the growth of current density, as shown in Equation (7). The fusing time decreased with the increase of current density. For small current densities, the melting time decreased with increasing wire length and with increasing wire diameter. The width of the oxide layer on the surface of the wire increased as the metal wire melts. The cross-sectional area of the current passing through the wire decreased and the local temperature increased, which accelerates the local melting. For small current density, when density current passes through the wire, the growth of the oxide layer on the surface is limited by the rapid decrease of Joule heat. For high current density, the coupling of surface oxidation and Joule heat determines the electric fusion of the wire.

### 4.5. Distinct Region of the Melted Wire

**Figure 14:**
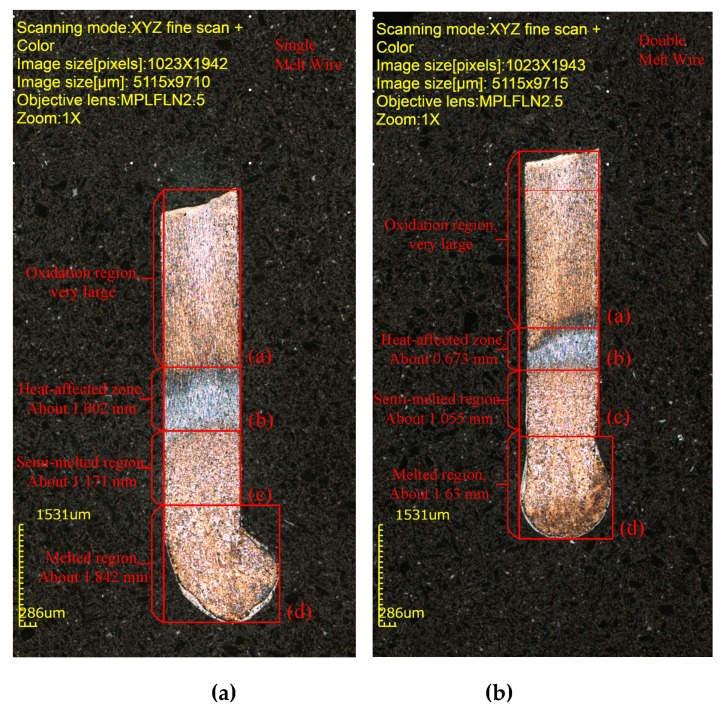
The laser scanning confocal microscope image of a melted wire. (**a**) Single metal wire and (**b**) double metal wire.

Figure 14 shows the laser scanning confocal microscope images of a molten metal wire. Figure 14a shows a micrograph of the fracture and solidification of a single metal wire during resistance heating, and Figure 14b is the micrograph of the double metal wire. The current density passing through the wire is 7.08 KA/mm^2^, the electrode distance is 8 mm, and the wire diameter is 1.2 mm. The heat melting to the solidification of wire can be divided into four different areas including oxidation area, heat-affected area, semi-melting area, and melting area. The length of the heat-affected zone, the semi-melted zone, and the melting zone of a single metal wire are 1.002 mm, 1.171 mm, and 1.842 mm, respectively. The lengths of the heat-affected zone, semi-melted zone, and melting zone of a double metal wire are 0.673 mm, 1.055 mm, and 1.63 mm. The distribution of Joule heat and heat dissipation in the melting process of single and double metal wire is different. The length of the heat-affected zone, the semi-melted zone, and the melting zone after the melting and solidification of a single metal wire is longer than the length of those zones after the double wire melting and solidification.

**Figure 15:**
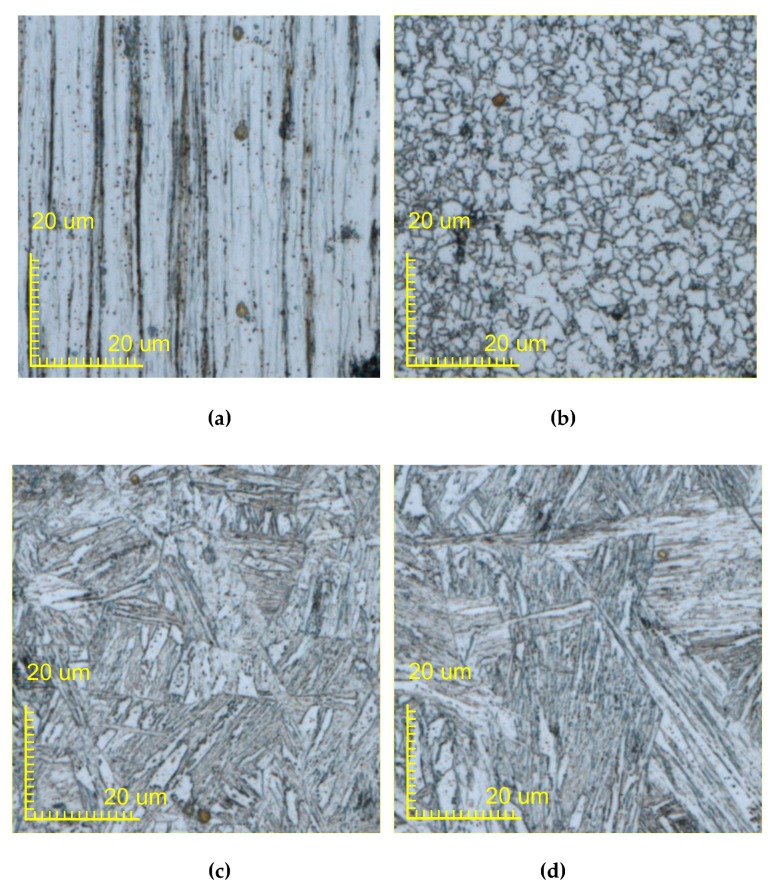
Micrograph of a single metal wire after melting and solidification. (**a**) The oxidation region, (**b**) the heat-affected zone, (**c**) the semi-melted region, and (**d**) the melted region.

**Figure 16:**
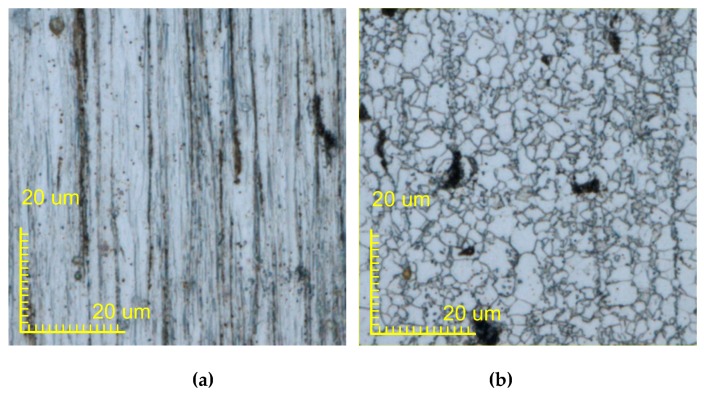

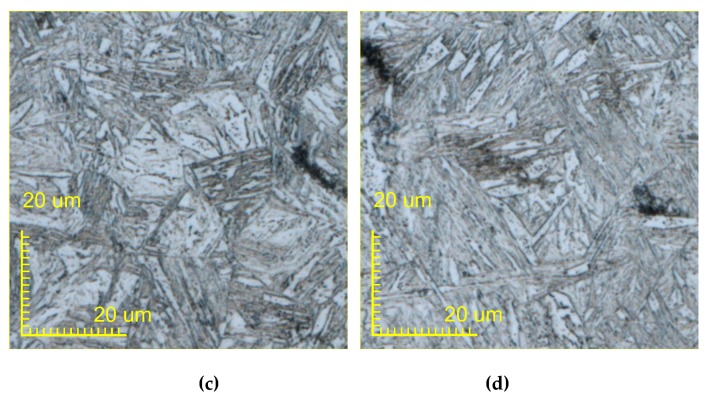
Micrographs of double metal wires after melting and solidification in (**a**) the oxidation region, (**b**) the heat-affected zone, (**c**) the semi-melted region, and (**d**) the melted region.

Figure 15 and Figure 16 show 200X micrographs of the single and double metal wire after melting and solidification, respectively. Figure 15a–d and Figure 16a–d show the microstructures of oxidation zone, heat affected zone, semi-melted zone, and melting zone of a single metal wire and the double metal wire, respectively. Figure 15a and Figure 16a show the microstructure in the oxidation region. Q235 wire is a product manufactured by the drawing process. This manufacturing process results in its microstructure becoming a unique band-shaped fine-grained ferrite and pearlite, which are fibrous deformation. Banded fine-grained ferrite and pearlite are relatively dense in some areas. In the oxidation area, due to the lack of sufficient heat energy to change, the structure of this area is roughly the same as the original structure of the wire. Figure 15b and Figure 16b show the microstructure of the heat-affected zone. The microstructure is a combination of ferrite grains being white and granular pearlite grains with block distribution. Black dots in the ferrite matrix are cementite particles. The gray grains at some grain boundaries are sulfide manganese inclusion. Black block is lamellar pearlite. Ferrite grain is small and evenly distributed. Because of low heating temperature and short holding time, the pearlite block has been transformed into austenite. Transformed into an acicular martensitic structure when it is cooled, ferrite has no transformation and is retained. After the temperature of the metal wire has approached the solid-liquid temperature, the wire begins to solidify. Figure 15c and Figure 16c show the microstructure in the semi-melted region with low-carbon martensite. Its microstructure is characterized by the same band-shaped martensite arranged in parallel, which forms martensitic bundles or martensite regions and results in a large positional difference between regions. Figure 15d and Figure 16d show the microstructure in the melted region. Due to the different degrees of decarburization of the wire, after heating, the ferrite structure is maintained in the decarburized layer. A small amount of low-carbon martensite appears in the ferrite, along with the troostite. The low carbon martensite has very little granular and un-melted ferrite. After the metal is melted and solidified, the microstructure consists of low carbon coarse martensite, and the sample was heated at a higher temperature to obtain coarse lath martensite.

The solidified structure after continuous melting and secondary contact melting is the same. Due to the different temperature distributions, the lengths of the melting regions are different. Therefore, the secondary contact melting of the melt tends to cause an uneven shape of the manufactured metal parts. By increasing the length of the metal wire, the temperature distribution can be changed, so that the melting length of the metal wire can be increased, and the problem of uneven shape of the part can be improved.

## 5. Conclusions

In this paper, the differences between the continuous melting process of the metal wire and the secondary contact melting process of the metal melt were analyzed. The results can be summarized as follows.

(1) The continuous melting and secondary contact melting of metal wire materials are different in the melting process in the solid-state temperature rising stage. There is a contact interface in the second contact of the melt, which can generate heat quickly. The temperature of the metal wire increased rapidly to 1100 °C, and then slowly increased to 1400 °C. When the metal wire is continuously melted, the temperature increases linearly to 1400 °C.

(2) The continuous melting and secondary contact melting of metal wire materials have different trends in dynamic resistance during a solid-state temperature rise. There is contact resistance during the second contact melting process. The resistance value decreased linearly to eliminate the contact interface, and then gradually increased the maximum value. During the continuous melting process of the metal wire, the resistance value increases linearly to the maximum value.

(3) The temperature distribution of the continuous melting process of the wire and the secondary contact melting process are different. The heat generated during the continuous melting process of the metal wire originates from the overall resistance of the metal wire, and the temperature distribution is “bow-shaped”. In the second contact melting, the heat generated by the contact interface resistance is transferred to both ends of the metal wire to melt, and the temperature distribution is “inverted V”.

(4) According to the temperature distribution and microstructure analysis of the metal wire, the microstructure distribution of the metal wire’s continuous melting and secondary contact melting solidification is the same. The continuous melting length of the metal wire is greater than the melting length of the secondary contact.

## Figures and Tables

**Table 1:** Experimental scheme of resistance heating wire.

Experimental Scheme	Current (A)	Current Waveform	Single/Double Metal Wire	Heating Length (mm)	Wire Diameter (mm)
1-1	60	Constant current	Single	4.0	1.2
1-2	80	Constant current	Single	4.0	1.2
1-3	100	Constant current	Single	4.0	1.2
2-1	60	Constant current	Single	4.0	1.2
2-2	60	Constant current	Single	6.0	1.2
2-3	60	Constant current	Single	8.0	1.2
3-1	60	Constant current	Single	4.0	0.8
3-2	60	Constant current	Single	4.0	1.2
3-3	60	Constant current	Single	4.0	1.6
4-1	60	Constant current	Double	2.0 and 2.0	1.2
4-2	60	Constant current	Double	3.0 and 3.0	1.2
4-3	60	Constant current	Double	4.0 and 4.0	1.2
